# Leprosy Reactions and Neuropathic Pain in Pure Neural Leprosy in a Reference Center in Rio de Janeiro – Brazil

**DOI:** 10.3389/fmed.2022.865485

**Published:** 2022-03-25

**Authors:** Izabela Jardim Rodrigues Pitta, Mariana Andrea Hacker, Robson Teixeira Vital, Ligia Rocha Andrade, Clarissa Neves Spitz, Anna Maria Sales, Sergio Luiz Gomes Antunes, Euzenir Nunes Sarno, Marcia Rodrigues Jardim

**Affiliations:** ^1^Leprosy Laboratory, Oswaldo Cruz Institute, Fiocruz, Rio de Janeiro, Brazil; ^2^Post-Graduate Program in Neurology, Federal University of the State of Rio de Janeiro, Rio de Janeiro, Brazil; ^3^Department of Neurology, Antonio Pedro University Hospital/ Fluminense Federal University, Niteroi, Brazil; ^4^Department of Neurology, Pedro Ernesto University Hospital/ Rio de Janeiro State University, Rio de Janeiro, Brazil

**Keywords:** leprosy, pure neural leprosy, leprosy reactions, neuritis, neuropathic pain

## Abstract

**Introduction:**

Leprosy reactions are complications that can occur before, during, or after multidrug therapy (MDT) and are considered a major cause of nerve damage. Neuritis is an inflammatory process that causes nerve function impairment associated with pain and tenderness along the nerve. Neuritis can be found in both type 1 and type 2 reactions and may also be the sole manifestation of a leprosy reaction. The objective of this study is to describe the incidence of leprosy reactions and its association with neuropathic pain in pure neural leprosy (PNL) patients.

**Methods:**

We selected 52 patients diagnosed with PNL and 67 patients with other clinical forms of leprosy. During the MDT the patients visited the clinic monthly to take their supervised dose. The patients were instructed to return immediately if any new neurological deficit or skin lesions occurred during or after the MDT.

**Results:**

Of the PNL patients, 23.1% had a leprosy reaction during or after the MDT, while this was 59.7% for patients with the other clinical forms of leprosy. There was an association between having PNL and not having any reaction during and after the MDT, as well as having PNL and having neuritis after the MDT.There was also an association between having previous neuritis and having neuropathic pain in the other clinical forms of leprosy group, although this association was not present in the PNL group.

**Discussion:**

Our data suggest that PNL is a different form of the disease, which is immunologically more stable. In addition, PNL patients have more neuritis than the classical leprosy skin reactions. In PNL there was no association between acute neuritis and neuropathic pain, suggesting that these patients may have had silent neuritis. Understanding and identifying neuritis is essential to reduce disability and the impact on public health.

## Introduction

Leprosy is usually known for its skin lesions, but neural abnormalities are another hallmark of the disease and the basis of leprosy-associated disability (1). Despite advances in the treatment of leprosy with the introduction of multidrug therapy (MDT) in the 1980s by the WHO, leprosy and its related disability are still prevalent worldwide (2).

Leprosy reactions are complications that can occur before, during, or after the MDT and are considered a major cause of nerve damage (3–6). They represent acute changes in the host immune response to *Mycobacterium leprae* and are thought to occur in 30–50% of all leprosy patients (2, 3). They are classically categorized in two subgroups according to the clinical and immunological presentation. Type 1 reactions usually have the development of an inflammatory response in the skin or nerves and are thought to occur in borderline leprosy patients, whose immunological status is unstable. Type 2 reactions are known to cause painful erythematous subcutaneous nodules as well as systemic symptoms, occur mainly in lepromatous leprosy, and are thought to be primarily humoral mediated (5, 6).

In the clinical evaluation, neuritis is an inflammatory process that affects the nerves and causes nerve function impairment associated with pain and tenderness along the nerve (3, 5, 6). It can be found in both type 1 and type 2 reactions and may also be the sole manifestation of a leprosy reaction, as an isolated neuritis without skin lesions (2, 4, 7–9). Nerve function impairment without pain and tenderness has been described as silent neuritis and may be present in a proportion of leprosy patients associated or not with skin reactional episodes (10). The presence of demyelinating features in nerve conduction studies (NCS) has already been described as occurring during neuritis episodes (6, 11, 12).

Silent or acute neuritis can result in nerve damage that may ultimately lead to the fearful disabilities (2). Neuropathic pain is characterized as a neural pain associated to dysfunction of the peripheral or central nervous sensory system that can be a consequence of neuritis (13). It has been estimated that there is an annual prevalence of 15% of neuropathic pain in leprosy patients and, although not usually included in the disability evaluation, this has a huge impact on the patients' quality of life (13).

There are few data regarding leprosy reactions in patients with pure neural leprosy (PNL), a rare form of the disease that presents with nerve function impairment without skin lesions (14). The objective of this study is to describe the incidence of leprosy reactions and its association with neuropathic pain in leprosy patients.

## Patients and Methodology

The patients in our study were selected from the database of the Souza Araujo Outpatient Clinic, at the Oswaldo Cruz Institute, a referral center for leprosy in Rio de Janeiro, Brazil. The study was approved by the Research Ethics Committee of the institution. We selected the 52 patients diagnosed with PNL between 1998 and 2016 that had neurological examination performed by a neurologist at the moment of diagnosis and at the end of the MDT, as well as NCS before the diagnosis. All of the PNL patients were evaluated by dermatologists who excluded the presence of skin lesions and were submitted to a nerve biopsy for diagnosis. The biopsied nerve was chosen accordingly to clinical and NCS impairment The methodology and criteria used for the histopathological diagnoses were those described by Antunes et al. (15). The PCR for *M. leprae* DNA and the detection of the antibodies against phenolic glycolipid-I (PGL-1) were done as the procedures described in Jardim 2003 and Jardim 2005, respectively (14, 16). All of the patients registered in the outpatient clinic in 2013 with the diagnosis of other clinical forms of leprosy were also selected. The diagnosis of these 67 patients was based on the positivity of slit-skin smear and skin biopsies, and the classification was made by a dermatologist according to the Ridley-Jopling system: tuberculoid (TT), borderline-tuberculoid (BT), borderline-borderline (BB), borderline-lepromatous (BL), lepromatous (LL), and undetermined. At the moment of the diagnosis the grade of disability of the patients was evaluated by a physiotherapist with experience in leprosy and based on WHO recommendations (17).

For treatment purposes, the patients with PNL were classified as either paucibacillary (PB) or multibacillary (MB) depending on the presence of bacilli in the slit-skin smears until 2005. After that, our reference center began to classify PNL patients as MB when acid-fast bacilli (AFB) were detected in the nerve biopsy.

During the MDT the patients had monthly visits to the clinic when they would take their supervised dose. A type 1 reaction was defined as an increased inflammation of existing lesions with or without new non-painful lesions and/or edema of the extremities. A type 2 reaction was defined as the sudden appearance of inflamed papules, nodules, and plaques sensitive to palpation. Leprosy reactions were always evaluated by a dermatologist and a type 1 or type 2 leprosy reaction was only considered in the presence of skin lesions. All of the patients with neural symptoms were evaluated by a neurologist and when neuritis was suspected the patient was submitted to NCS using the procedures described by Vital et al. (18). The patient was considered as having neuritis when there was pain and tenderness of the nerve together with demyelinating features like reduced motor conduction velocities, presence of conduction block, or abnormal temporal dispersion. The patients were instructed to return immediately in the case of any new neurological deficit or skin lesions during or after the MDT. All of the patients with leprosy reactions were treated with corticosteroids. Neuropathic pain was always evaluated by a neurologist, and its diagnosis was based on the presence of pain in a neuro-anatomically plausible area with confirmed negative or positive sensory signs (13).

Statistical analysis was performed by the Pearson's chi-square and Fisher's exact tests for categorical variables and Mann-Whitney test for continuous variables using the *Statistical Package for the Social Sciences* (SPSS) v16.0. A significance level of 5% was adopted.

## Results

For this study, 52 patients with PNL and 67 patients with other clinical forms of leprosy were selected. Of the PNL patients, 98.1% (51 patients) were treated with PB-MDT and 1.7% (1 patient) were treated with MB-MDT. In the other clinical forms group, 50.7% (34 patients) were treated with PB-MDT and 49.3% (33 patients) with MB-MDT. In the PNL group, the mean age of patients at diagnosis was 47 years and the median was 46 years; while in the other clinical forms group, the mean age of the patients was 42 years and the median was 39 years (*p* = 0.086). In the PNL group, 73.1% (38 patients) were male and 26.9% (14 patients) were female; in the other clinical forms group, 58.2% (39 patients) were male and 41.2% (28 patients) were female (*p* = 0.097). The patients with other clinical forms were classified as follows: 3% as TT (2 patients); 43.3% as BT (29 patients); 10.4% as BB (7 patients); 19.4% as BL (13 patients); 20.9% as LL (14 patients); and 3% as undetermined. The mean follow-up period for the PNL group was 188 months (ranging from 47 to 260 months) and for the other clinical forms group the mean follow-up period was 93 months (ranging from 89 to 98 months).

### Reactional Episodes

Patients with PNL and those with other clinical forms of leprosy were compared in relation to the presence of reactions during and after the MDT. Of the PNL patients, 12 (23.1%) had a leprosy reaction during or after the MDT, while 40 patients (59.7%) of the other clinical forms group had a leprosy reaction. Pearson's chi-square confirmed that there was an association of having other clinical forms of leprosy and having leprosy reactions during or after the MDT (*p* < 0.001). The greater incidence of reactions during or after the MDT was present for the BB, BL and LL forms (*p* < 0.001). When comparing the PNL group with the TT and BT groups there was no difference in the incidence of leprosy reactions (*p* = 0.606). The number of patients in each group during the MDT is shown in Table 1 and after the MDT in Table 2.

**Table 1:** Number of patients with pure neural leprosy and other clinical forms with leprosy reactions and neuritis during the multidrug therapy.

		**PNL**	**Other clinical forms**	**Total**
No leprosy reaction		**46 (88.5%)**	33 (49.3%)	79
Leprosy reaction	Type 1 reaction	2 (3.8%)	**14 (20.9%)**	16
	Type 2 reaction	0	7 (10.4%)	7
	Neuritis	**3 (5.8%)**	2 (3.0%)	5
	Type 1 reaction with neuritis	1 (1.9%)	7 (10.4%)	8
	Type 2 reaction with neuritis	0	4 (6.0%)	4
	Total	52	67	119

*PNL, Pure neural leprosy*.

**Table 2:** Number of patients with pure neural leprosy and other clinical forms with leprosy reactions and neuritis after the multidrug therapy.

		**PNL**	**Other clinical forms**	**Total**
No leprosy reaction		**45 (86.5%)**	45 (67.2%)	90
Leprosy reaction	Type 1 reaction	0	6 (9.0%)	6
	Type 2 reaction	0	**9 (13.4%)**	9
	Neuritis	**6 (11.5%)**	0	6
	Type 1 reaction with neuritis	1 (1.9%)	5 (7.5%)	6
	Type 2 reaction with neuritis	0	2 (3.0%)	2
	Total	52	67	119

*PNL, Pure neural leprosy*.

Fisher's exact test confirmed that there was association between having PNL and not having any reaction and an association between having other clinical forms of leprosy and having type 1 and type 2 reactions during and after the MDT (*p* < 0.001). Fisher's exact test also confirmed an association between having PNL and having neuritis after the MDT (*p* < 0.001). There was no association between any clinical form and having type 1 and type 2 reactions together with neuritis.

### Neuropathic Pain

At the moment of leprosy diagnosis, in the PNL group 11.5% (6 patients) had neuropathic pain, while this was only 2.9% (two patients) for the other clinical forms group. Despite this, Fisher's exact test did not show statistically significant association between the clinical form and the presence of neuropathic pain at diagnosis (*p* = 0.078).

The mean time between the beginning of the MDT and the emergence of neuropathic pain was 32 months and the median was 8 months (min 0, maximum 156 months) in the PNL group. In the other clinical forms group, the mean period was 12 months and the median was 8 months (minimum 0, maximum 53 months) (*p* = 0.254).

In terms of neuropathic pain, 13 patients (25%) in the PNL group and 13 patients (19.2%) in the other clinical forms group presented this after the MDT. In the PNL group, Fisher's exact test confirmed an statistically significant association between having more than one nerve affected at neurological examination and developing neuropathic pain (*p* = 0.044). Fisher's exact test confirmed an association between having previous neuritis during or after the MDT and having neuropathic pain in the other clinical forms of leprosy group. The association was not present in the PNL group. The number of patients with neuritis and the patients that developed neuropathic pain in both groups are reported in Table 3.

**Table 3:** Number of patients with pure neural leprosy and other clinical forms of leprosy that developed acute neuritis during the evaluation period and number of patients that developed neuropathic pain.

			**Acute neuritis**		***p*-value**
			**No**	**Yes**	
PNL	Neuropathic pain	No	32 (61.5%)	7 (13.4%)	0.697
		Yes	**10 (19.2%)**	3 (5.7%)	
Other clinical forms	Neuropathic pain	No	49 (73.1%)	5 (7.4%)	<0.001
		Yes	5 (7.5%)	**8 (11.9%)**	

*PNL, Pure neural leprosy*.

### Histopathological Correlations

When evaluating the nerve biopsies of the PNL group that were made for the diagnosis, 7.6% (four patients) had the presence of AFB and epithelioid granuloma. Fisher's exact test did not show statistically significant association between these histopathological signs and neuritis during or after the MDT (*p* = 1.000 and *p* = 0.450) or with neuropathic pain at diagnosis (*p* = 1.000).

All of the PNL patients and 62 of the patients with other clinical forms had their degree of leprosy-related disability evaluated at diagnosis. Of these, 41 (78.8%) of the PNL patients and 25(40.3%) of the patients with other clinical forms had disabilities. Person's chi-square confirmed an association between having PNL and having any degree of disability (*p* < 0.001).

## Discussion

Leprosy reactions are inflammatory episodes related to changes in the immune response of the patients and are thought to be one of main causes for nerve damage in leprosy (2). PNL is a form of the disease characterized by the absence of skin lesions and a negative slit-skin smear (14, 19). PNL is a still poorly understood form of the disease and there are few data regarding the presence of leprosy reactions in these patients.

Leprosy reactions are present in 30–50% of all leprosy patients and can present in any moment of the disease (2, 8). In our sample, the groups with other clinical forms of leprosy had a frequency of leprosy reactions within the range that is described in the literature. However, the frequency was much lower in the PNL group, which could suggest that this is a different form of the disease. This difference was only observed when specifically comparing the PNL group to the BB, BL, and LL patients. This data could suggest that PNL patients may be similar to the patients in the tuberculoid pole, the TT and BT patients. The classification system proposed by Ridley and Jopling is based on the immune response to the bacilli and does not include PNL (20). This greater immune response to the bacilli could explain the fact that most PNL patients, despite having neuropathy symptoms for a long time before diagnosis, do not have episodes of pain and tenderness along the nerve. A type 2 leprosy reaction is usually observed in patients in the lepromatous pole of the disease and associated with a higher burden of bacilli in slit-skin smear (2, 8). None of the patients in the PNL group had type 2 leprosy reactions, even when they had more extension of nerve lesions.

Both type 1 and type 2 reactions are associated with nerve damage and neuritis, but neuritis can also appear alone as an isolated neuritis (2, 4, 7–9). In our sample, in the other clinical forms of leprosy, neuritis alone was an uncommon finding, but in the PNL group it was more common than the neuritis associated with a type 1 reaction. It has been described that genetic variability may be responsible for the variable clinical phenotypes of leprosy (1, 2). Our data suggest that the reactional episodes in PNL patients are also limited to the peripheral nerve. It is not known what causes the disease to stay restricted to the nerve at presentation, but the same mechanisms may be responsible for this in the reactional episodes. It has also been described that while the MDT is capable of killing the *M. leprae*, it still leaves dead bacterial cells within the nerve (7). These fragments may be responsible for triggering new episodes of neuritis, especially in the PNL group where the disease was present only in the nerve.

The term neuritis has been used to describe nerve function impairment associated with nerve pain and tenderness associated to demyelinating features in the NCS (3, 6, 11, 12). However, the existence of silent neuritis, where the patient has nerve function impairment without pain, has been well-described in the leprosy literature (10). The majority of patients in our sample did not present clinical signs and symptoms of acute neuritis. If we consider that all of the PNL patients have nerve function impairment caused by the disease, associated or not with nerve pain and tenderness, we could suggest that the majority of these PNL diagnosed patients may have had silent neuritis prior to the diagnosis.

Neuropathic pain is described as increased pain sensitivity or spontaneous pain caused by lesions or diseases involving the somatosensory system (21). In our sample, in both groups, neuropathic pain was more common after the MDT, as was previously suggested by other authors (22). Our data also suggest that in the other clinical forms of leprosy group there is an association between previously having neuritis and having neuropathic pain, since these patients were diagnosed based on skin lesions and most of them did not have any neural symptom prior to the diagnosis. This is unlike the patients with PNL, who have nerve function impairment and therefore may be susceptible to neuropathic pain.

The prevalence of neuropathic pain in our PNL group was lower than the 60% prevalence described in diabetic neuropathy (23). However, the neuropathy severity is thought to be one of the risk factors for neuropathic pain in diabetic neuropathy (23). The statistically significant association between having more than one nerve clinically affected and developing neuropathic pain may also suggest that this may also be true in PNL. Since most of our PNL patients have a small number of affected nerves, this could be one of the explanations for the lower prevalence of neuropathic pain.

It has been suggested that the presence of both AFB and epithelioid granuloma in the same biopsy specimen may indicate reactional neuritis (15, 24). In our PNL patient group, only 7.6% had these features in the nerve biopsy, which can be explained by the fact that they did not have acute neuritis at the moment when the biopsy was conducted. The lack of association between the histopathological neuritis and the presence of neuropathic pain at the diagnosis may be considered another sign that acute neuritis is not the only mechanism involved in the generation of neuropathic pain in PNL.

Although leprosy reactions are thought to be one of the greatest causes for disability in leprosy (2, 3), patients with PNL had a greater burden of disability than the other clinical forms despite the lowest incidence of leprosy reactions. This could be explained by the fact that in all of these patients the *M. leprae* initially targets the Schwann cell within the peripheral nerve. Although the host immune response has a critical role in the neural damage in leprosy, it has already been described that the *M. leprae* itself may initiate nerve damage, even in the absence of the host inflammatory response (1, 7).

The term neuritis has been used in numerous ways in the leprosy literature, sometimes describing the acute episode, as in a leprosy reaction, and sometimes as the silent nerve impairment caused by the disease (13). Limitations are present in this study, including the fact that the data is retrospective. Nevertheless our study showed that in PNL during reactional episodes, acute neuritis is usually easily diagnosed, but silent neuritis is still under recognized. The identification and understanding of silent neuritis is of great importance as it may help reduce the physical disability, and could also the pain-related disability that may have economic and social impacts.

## Data Availability Statement

The original contributions presented in the study are included in the article/supplementary material, further inquiries can be directed to the corresponding author/s.

## Ethics Statement

The studies involving human participants were reviewed and approved by Research Ethics Committee of the Oswaldo Cruz Foudation (Fiocruz). Written informed consent for participation was not required for this study in accordance with the national legislation and the institutional requirements.

## Author Contributions

IP: conceptualization, data curation, investigation, formal analysis, and writing. MH: conceptualization, data curation, formal analysis, and writing. RV, LA, CS, AS, and SA: investigation. ES: conceptualization and investigation. MJ: conceptualization, investigation, writing, and supervision. All authors contributed to the article and approved the submitted version.

## Conflict of Interest

The authors declare that the research was conducted in the absence of any commercial or financial relationships that could be construed as a potential conflict of interest.

## Publisher's Note

All claims expressed in this article are solely those of the authors and do not necessarily represent those of their affiliated organizations, or those of the publisher, the editors and the reviewers. Any product that may be evaluated in this article, or claim that may be made by its manufacturer, is not guaranteed or endorsed by the publisher.
